# Auditory modulation of visual stimulus encoding in human retinotopic cortex

**DOI:** 10.1016/j.neuroimage.2012.12.061

**Published:** 2013-04-15

**Authors:** Benjamin de Haas, D. Samuel Schwarzkopf, Maren Urner, Geraint Rees

**Affiliations:** UCL Institute of Cognitive Neuroscience, 17 Queen Square, London WC1N 3BG, UK; Wellcome Trust Centre for Neuroimaging, University College London, 12 Queen Square, London WC1N 3AR, UK

**Keywords:** Multisensory, Audio-visual, V2, Decoding, MVPA, fMRI

## Abstract

Sounds can modulate visual perception as well as neural activity in retinotopic cortex. Most studies in this context investigated how sounds change neural amplitude and oscillatory phase reset in visual cortex. However, recent studies in macaque monkeys show that congruence of audio-visual stimuli also modulates the amount of stimulus information carried by spiking activity of primary auditory and visual neurons. Here, we used naturalistic video stimuli and recorded the spatial patterns of functional MRI signals in human retinotopic cortex to test whether the discriminability of such patterns varied with the presence and congruence of co-occurring sounds. We found that incongruent sounds significantly impaired stimulus decoding from area V2 and there was a similar trend for V3. This effect was associated with reduced inter-trial reliability of patterns (i.e. higher levels of noise), but was not accompanied by any detectable modulation of overall signal amplitude. We conclude that sounds modulate naturalistic stimulus encoding in early human retinotopic cortex without affecting overall signal amplitude. Subthreshold modulation, oscillatory phase reset and dynamic attentional modulation are candidate neural and cognitive mechanisms mediating these effects.

## Highlights

► Multivariate decoding of video identity from fMRI signals in V1V3. ► Decoding accuracy in V2 is significantly reduced for incongruent sounds. ► Reduced decoding accuracy is associated with reduced inter-trial reliability. ► No modulation of univariate signal amplitude by sounds. ► Noise levels in sensory areas are affected by multisensory congruence.

## Introduction

Perception of the environment requires integration of sensory information across the senses, but how our brains combine information from different sensory streams is still poorly understood. The earliest stages of cortical sensory processing were long thought to be unimodal and multisensory processing to be restricted to dedicated convergence areas (Mesulam, 1998). However, the past decade has seen new anatomical and functional evidence for multisensory interactions even at the level of primary sensory areas (see Driver and Noesselt, 2008; Klemen and Chambers, 2012 for an overview).

Tracer studies provide anatomical evidence for multisensory interactions at early stages of cortical processing (here referred to as ‘early multisensory interactions’ for convenience, not necessarily implying temporal precedence). There are direct feedback connections from primary auditory and multisensory areas to V1 and V2 in macaque (Clavagnier et al., 2004; Falchier et al., 2002; Rockland and Ojima, 2003) and similar connections in rodents (Allman et al., 2008; Budinger et al., 2006). Although some bimodal neurons can be found even in primary sensory areas (i.e. neurons that can be driven by either visual or auditory input, e.g. Fishman and Michael, 1973), the effect of direct cross-modal connections seems to be modulatory, rather than driving. Recent evidence from cats and rodents points to subthreshold modulation of ‘unimodal’ visual neurons (that cannot be driven by auditory input alone) as the dominant form of multisensory interaction in early visual cortex (Allman and Meredith, 2007; Allman et al., 2008, 2009; Iurilli et al., 2012). Early multisensory interactions also result in phase resetting of ongoing oscillations, thereby modulating and aligning the periodic excitability of affected neurons (e.g. Lakatos et al., 2007, 2009, cf. Schroeder et al., 2008).

In humans, cross-modal interactions modulate the amplitude or can drive neural signals in early visual cortex, as indexed by Blood Oxygenation Level Dependent (BOLD) fMRI (e.g. Macaluso et al., 2000; Martuzzi et al., 2007; Meienbrock et al., 2007; Noesselt et al., 2007; Watkins et al., 2006), event-related potentials (ERPs) (e.g. Cappe et al., 2010; Molholm et al., 2002) and transcranial magnetic stimulation (TMS) excitability (e.g.Romei et al., 2009). Cross-modal phase reset of ongoing oscillations in visual cortex is found in human magnetoencephalography (MEG; Luo et al., 2010) and electroencephalography (EEG; consistent with phase-locked periodic modulations of perceptual performance Naue et al., 2011; Romei et al., 2012; Thorne et al., 2011).

When monkeys are presented with naturalistic sound stimuli, accompanying visual stimulation reduces the mean firing rate of primary auditory cortex neurons (Dahl et al., 2010; Kayser et al., 2010). Moreover, inter-trial variability of spike trains is greatly reduced, thus enhancing mutual information between stimuli and spiking patterns. This effect is significantly stronger when the auditory and the visual input are congruent (Kayser et al., 2010). Visual neurons in STS show a similar behaviour for naturalistic visual stimuli (Dahl et al., 2010). Their response amplitude is somewhat reduced for bimodal audio-visual stimulation and the stimulus information carried by spike patterns is affected by multisensory context: incongruent sounds significantly worsen stimulus decoding based on spike trains.

Here we sought to test whether multisensory modulation of stimulus encoding extended to humans and early retinotopic visual cortices. We presented participants with naturalistic audiovisual stimuli in four different conditions: audio only (A), visual only (V), audiovisual congruent (AV congruent) and audio-visual incongruent (AV incongruent). We then used multivoxel pattern analysis (MVPA) to decode stimulus identities based on spatial patterns of BOLD signals evoked in V1-3 (as identified by retinotopic mapping, Sereno et al., 1995). Separate multivariate classifiers were trained and tested for each of the four conditions and for each ROI. This allowed us to compare decoding accuracies between conditions, thus obtaining an index of pattern discriminability for each condition.

## Materials and methods

### Participants

15 participants from the University College London (UCL) participant pool took part (mean age, 26 years, SD, 4 years; 7 females; 1 left handed). All participants had normal or corrected to normal vision and reported no hearing problems. Written informed consent was obtained from each participant and the study was approved by the UCL ethics committee. Participants were paid 10 GBP per hour for taking part in the experiment, which lasted up to 2.5 h.

### Stimuli

Four video clips were used as audio-visual stimuli, each lasting 3 s. Two clips showed natural scenes containing animals (a croaking frog and a crowing rooster). These two clips were downloaded from http://www.youtube.com and edited. The two remaining clips showed the clothed torso of the first author while turning a key in a lock or ripping a paper apart. All clips were similar with regard to luminance and loudness and were projected onto a screen at the end of the scanner bore. Participants viewed the clips via a mirror mounted at the head coil of the scanner at a viewing distance of ~ 72 cm. Video clips were presented at a resolution of 640 × 360 pixels and subtended ~ 18 by 10° visual angle when viewed by participants in the scanner. During the experiment participants were asked to fixate a white dot projected on top of the videos at the centre of the screen (radius ~ 0.1° visual angle). In each trial the dot turned blue once, twice or three times and participants were asked to count and indicate the number of colour changes via a button box in a 2 s inter stimulus interval.

Audio tracks accompanying the video clips were presented via MRI compatible in-ear headphones (http://www.etymotic.com). Loudness was adjusted individually before the start of the experiment, aiming for a level that was comfortable for participants but still enabled them to easily tell apart sound clips in the presence of scanner noise.

All stimuli were programmed and presented in MATLAB (Mathworks, Ltd.) using the Cogent Graphics (http://www.vislab.ucl.ac.uk/cogent.php) and Psychophysics Toolbox 3 extensions (Brainard, 1997; Pelli, 1997; http://psychtoolbox.org).

### Procedure

**Fig. 1:**
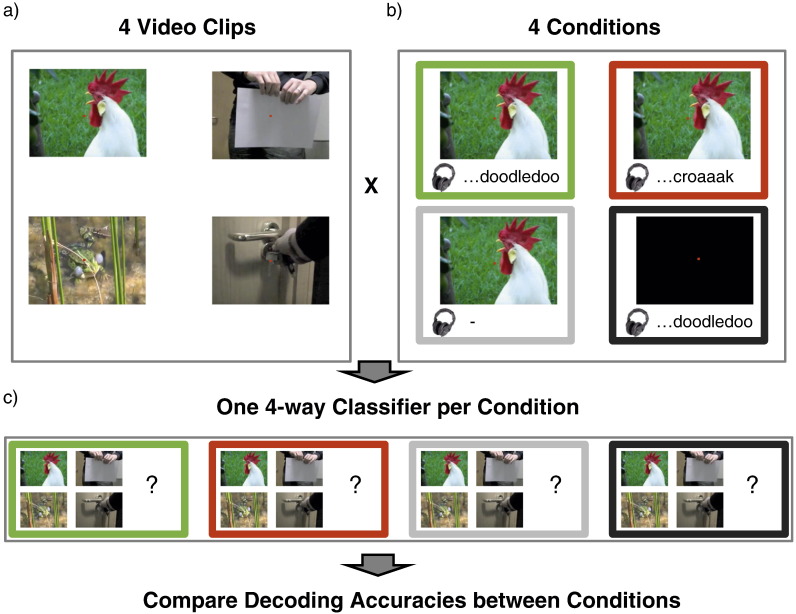
Design. a)Four audiovisual clips used as stimuli, each lasting 3 s. Participants counted colour changes of the fixation dot in each trial.b)Each of the clips was presented multiple times in four conditions (illustrated here for one example clip): audiovisual congruent (AV congruent) in green, audiovisual incongruent (AV incongruent) in red, visual only (V) in light grey and audio only (A) in dark grey.c)Separate multivariate classifiers were trained to decode which of the four stimuli was presented for each condition.

Each participant completed 17–24 runs of scanning in the main experiment, each run lasting just under 2 min. During the runs participants were presented with audio and/or visual clips and completed an incidental, superimposed fixation task (cf. above). During each run each of the 4 stimuli was presented once for each experimental condition (i.e. four times), amounting to 16 stimulus trials per run (cf. Fig. 1). Participants were either presented with videos only (V), sounds only (A), matching videos and sounds (AV congruent condition) or mismatching videos and sounds (AV incongruent condition). For audio-visually incongruent trials the sound files were swapped between fixed pairs of videos (rooster crowing and paper ripping; frog croaking and keys turning). Each 3 s clip was followed by a 2 s inter-stimulus interval during which participants were asked to indicate via a button box how many times the fixation dot changed its colour. In addition to the 16 stimulus trials there were 4 blank trials in each run that served as a baseline measure. During these trials participants completed the fixation task in the absence of audio-visual clips. The order of the 20 trials was randomised for each run, as was the number of fixation dot colour changes in each trial (1–3).

### Retinotopic mapping

To delineate the borders of visual areas V1-3 on an individual basis, each participant underwent an additional fMRI run viewing stimuli for phase encoded retinotopic mapping (Sereno et al., 1995). Stimuli for this run consisted of a wedge rotating clock-wise and an expanding ring. Both stimuli moved in discrete steps, synchronised with the acquisition of fMRI volumes, but with different frequencies (wedge: 12 cycles, 20 steps per cycle; ring: 20 cycles, 12 steps per cycle). They were centred around a fixation dot of ~ 0.25° diameter and spanned up to 8° of eccentricity. It is generally difficult to distinguish retinotopic maps inside the foveal confluence because the borders between regions are difficult to resolve at conventional voxel sizes. Moreover, the presence of a stable fixation dot precludes any systematic variation in the BOLD signal related to the mapping stimulus. Note that the size of the fixation dot for our mapping stimuli was slightly larger than the size of the fixation dot for our audiovisual stimuli (~ 0.25 vs. ~ 0.2° diameter). We are therefore confident that our region of interest analyses did not include the foveal representations. Ring and wedge were presented on a grey background and served as apertures revealing a dynamic high contrast stimulus. Participants were asked to fixate at all times and count brief colour changes of the fixation dot from blue to purple. These colour change events lasted 200 ms and could occur at every non-consecutive 200 ms window of the run with a probability of 5%.

### Image acquisition and pre-processing

All functional and structural scans were obtained with a Tim Trio 3T scanner (Siemens Medical Systems, Erlangen, Germany), using a 12-channel head coil. Functional images for the main experiment were acquired with a gradient echo planar imaging (EPI) sequence (3 mm isotropic resolution, matrix size 64 × 64, 40 transverse slices per volume, acquired in ascending order (whole head coverage); slice acquisition time 68 ms, TE 30 ms, TR 2.72 s). We obtained 42 volumes per run of the main experiment (including three dummy volumes at the beginning of each run and two at the end), resulting in a run duration of 114.24 s. Functional images for retinotopic mapping were acquired in one run of 247 volumes with an EPI sequence (including five dummy volumes at the beginning and two at the end of the run; 2.3 mm isotropic resolution, matrix size 96 × 96, 36 transverse slices per volume, acquired in interleaved order (centred on the occipital cortex); slice acquisition time 85 ms, TE 36 ms, TR 3.06 s per volume). In between the main experiment and the retinotopic mapping run we acquired fieldmaps to correct for geometric distortions in the functional images caused by heterogeneities in the B0 magnetic field (double-echo FLASH sequence with a short TE of 10 ms and a long sequence of 12.46 ms, 3 × 3 × 2 mm, 1 mm gap). Finally, we acquired a T1-weighted structural image of each participant using an MDEFT sequence (Deichmann et al., 2004; 1 mm isotropic resolution, matrix size 256 × 240, 176 sagittal slices, TE 2.48 ms, TR 7.92 ms, TI 910 ms).

All image files were converted to NIfTI format and pre-processed using SPM 8 (http://www.fil.ion.ucl.ac.uk/spm/software/spm8/). The dummy volumes for each run were discarded to allow for the T1 signal to reach steady state. The remaining functional images of the main experiment and the retinotopic mapping session were independently mean bias corrected, realigned and unwarped (using voxel displacement maps generated from the fieldmaps). Finally the functional images were co-registered with the respective anatomical MDEFT scan for each participant and smoothed with a 5 mm Gaussian kernel.

### Data analysis

#### Multivariate pattern analysis

We specified separate general linear models for each run and each participant. Each general linear model contained regressors for each of the 16 trial types plus one regressor for the blank trials (boxcar regressors convolved with a canonical hemodynamic response function). Additional regressors of no interest were modelled for response intervals and for the six motion parameters estimated during re-alignment. The general linear models for each run and each participant were estimated and contrast images for each of the 16 trials (per run and condition) calculated. This resulted in separate contrast images and t-maps for each trial type of the experiment for each participant. These t-maps were masked with the retinotopic regions of interest (see below) and the resulting patterns were vectorised. For the decoding and correlation analyses the resulting patterns were mean corrected across stimuli within each condition. Note that this did not affect classification performance — the distribution of patterns in feature space was preserved, but now centred on zero. This allowed us to ensure that any common intercept of patterns across stimuli was disregarded for the similarity and reliability correlation analyses (see below). Beta maps for univariate analyses were not mean corrected. The aim of the decoding analysis was to decode *stimulus identity* from activation patterns in visual areas (i.e. which of the four videos was presented in a given trial) and to compare the accuracies of decoders *across conditions* (i.e. did stimulus decoding accuracy vary depending on audiovisual condition, cf. Fig. 1). Stimulus decoding was performed using custom code using the linear support vector machine (lSVM) implemented in the Bioinformatics toolbox for MATLAB (version R2010b, http://www.mathworks.com). Data from each condition were used for training and testing of separate classifiers to get condition-specific decoding accuracies. For each condition a four-way classifier was built, to decode which of the four stimuli was presented from a given activation pattern. The four-way classifier consisted of six lSVMs to test all possible pair-wise comparisons between the four stimuli. It then assigned one of the stimulus labels based on a one-against-one voting procedure (Hsu and Lin, 2002). The four-way classifier was trained and tested for accuracy in a jackknife procedure. In each iteration, the (condition-specific) data from all runs but one served as training data and the (condition-specific) data from the remaining run was used to test the prediction accuracy of the lSVM. Accuracies were stored and averaged across iterations at the end of this procedure, and the whole procedure was applied to each retinotopic ROI (V1-3) independently, yielding a four-way classification accuracy for each condition and ROI. Statistical analysis of the resulting accuracies was done in MATLAB and PASW 18.0 (SPSS inc./IBM). Accuracies were compared against chance level by subtracting .25 and using one sample t-tests. Accuracies were compared between conditions using ANOVAs and paired t-tests.

Potential differences in decoding accuracy between conditions could stem from two different sources. They could be due to changes in pattern reliability across trials, changes in pattern similarity between patterns evoked by different stimuli or both. We employed additional analyses to differentiate between those options. Changes in pattern reliability were tested by averaging the patterns for a given stimulus across trials separately from odd and even runs and computing the Pearson correlation coefficient for the two resulting mean patterns (in a ROI-and condition-specific manner). The resulting correlation coefficients were Fisher z-transformed, averaged for each condition and then compared across conditions using ANOVAs and paired t-tests. Changes in pattern similarity were tested by averaging the patterns for a given stimulus across all trials and computing correlations between these mean patterns for different stimuli (again, in a ROI- and condition-specific manner). The resulting Pearson correlation coefficients were compared as described above.

#### Searchlight analysis

To test whether and where stimulus information was modulated by audiovisual context outside retinotopic cortices, we set up an additional, exploratory searchlight analysis (Kriegeskorte et al., 2006). For this analysis, activation patterns were derived from the same (trial-specific) t-maps that were used for the ROI analysis described above. The searchlight consisted of a sphere with a radius of 4 voxels that was centred on each grey matter voxel of each participant's brain in turn. During each iteration, the searchlight was used as a mask and the patterns of activation within this mask were read out for each trial. Then the same 4-way classification procedure used for the ROI analysis was applied to those patterns (cf. above). The resulting (condition specific) classification accuracies were projected back onto the seed voxel. Repeating this procedure for every grey matter voxel, we thus derived four accuracy maps for each participant (one per condition). To test for significant accuracy differences between conditions we subtracted the respective accuracy maps from each other. Specifically, we contrasted the audiovisual congruent condition with the muted condition and with the incongruent condition and the muted condition with the audio-visual incongruent condition. The resulting accuracy contrast maps were normalised to MNI space (http://www.loni.ucla.edu/ICBM/) and tested for whole brain family-wise error (FWE) corrected significance at cluster level in SPM 8 (cluster forming threshold *p* < .001 uncorrected). Significant clusters were identified anatomically using the Juelich Histological Atlas implemented in the SPM Anatomy Toolbox (v. 1.8, http://www.fz-juelich.de/inm/inm-1/DE/Forschung/_docs/SPMAnatomyToolbox/SPMAnatomyToolbox_node.html).

#### Univariate analysis

To test whether audio-visual context had any influence on the overall signal amplitude in our ROIs we employed an additional univariate analysis. For this analysis we averaged the condition specific beta weights of voxels within our ROIs across stimuli and trials for each participant. We then compared the mean beta values between conditions for each ROI using ANOVAs and paired t-tests.

We additionally tested whether a different approach to univariate analyses would have yielded any differences between conditions. To test this, we concatenated all runs of a given participant in one design matrix in SPM8. This allowed us to build contrasts between conditions on the first level, utilising all trials of the respective conditions. These first level contrasts were then normalised to MNI space and tested for whole brain FWE corrected significance at cluster level in SPM8 (cluster forming threshold *p* < .001 uncorrected).

#### Retinotopic mapping

Retinotopic ROIs were identified using standard phase-encoded retinotopic mapping procedures (Sereno et al., 1995). We extracted and normalised the time series for each voxel and applied a fast Fourier transformation to it. Visually responsive voxels were identified by peaks in their power spectra that corresponded to our stimulus frequencies. The preferred polar angle and eccentricity of each voxel was then identified as the phase lag of the signal at the corresponding stimulus frequency (wedge and ring, respectively). The phase lags for each voxel were stored in a ‘polar’ and an ‘eccentricity’ volume and then projected onto the reconstructed, inflated cortical surface (surface based analysis was performed using FreeSurfer: http://surfer.nmr.mgh.harvard.edu). The resulting maps allowed us to identify meridian polar angle reversals and thus to delineate the borders of visual areas V1-3 on the cortical surface. These labels were then exported as three-dimensional masks into NIfTI space and served as ROIs.

## Results

### Behavioural data

Participants performed well on the fixation task for all four stimulus categories and the baseline category. Performance did not differ significantly between conditions (note that the task was independent of stimulus category; 95 ± 1%, 96 ± 1%, 96 ± 1%, 97 ± 1%, and 97 ± 1% correct for the AV congruent, AV incongruent, V, A and baseline category, respectively (mean ± standard error of the mean); *F*_(2.49, 34.85)_ = 1.59, *p* = .22, *n.s.*, Greenhouse–Geisser corrected for non-sphericity).

### Multivariate fMRI results

#### Multivariate ROI results

**Fig. 2:**
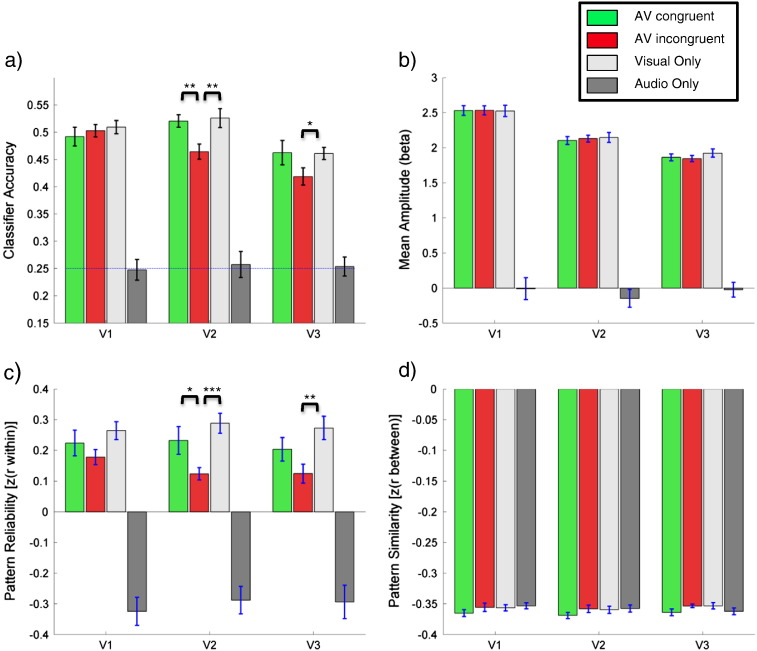
Results for regions of interest (ROIs). Results for areas V1-3 are shown as bar plots. Bar colours indicate conditions: audiovisual congruent in green, audiovisual incongruent in red, visual only in light grey and audio only in dark grey. Error bars indicate the standard error of the mean adjusted for repeated measurements (Morey, 2008). a)Classification accuracies for 4-way classification using linear support vector machines (see Materials and methods for details). The dashed line indicates chance level (.25). Stars indicate significantly different decoding accuracies between conditions involving visual stimulation (as indicated by paired *t*-tests, see Results section for details of respective ANOVAs; *p < .05, **p < .01).b)Mean signal amplitudes estimated by the GLM. Note that amplitudes were not significantly different between conditions involving visual stimulation in any of the regions of interest. Note that beta maps used for this analysis were not mean corrected (see Materials and methods for details).c)Pattern reliability as indicated by means of Fischer z-transformed correlation coefficients between patterns for a given stimulus in odd and even runs (see Materials and methods for details). Stars indicate significantly different pattern reliabilities between conditions involving visual stimulation (as indicated by paired *t*-tests, see Results section for details of respective ANOVAs; *p < .05, **p < .01, ***p < .001).d)Pattern similarity as indicated by means of Fischer z-transformed correlation coefficients between patterns for different stimuli (see Materials and methods for details). Note that pattern similarities were not significantly different between conditions involving visual stimulation in any of the regions of interest. Patterns are negatively correlated because they were mean corrected across stimuli within each condition (see Materials and methods for details).

Visual stimulus identities could be decoded significantly above chance level (0.25) from V1-3 (ROIs were combined across hemispheres; all *p* < 10^− 5^, cf. Fig. 2a)). When no visual stimulus was presented (A condition) decoding performance was at chance level (all *p* > .4). To test whether the presence and congruence of co-occurring sounds had an influence on visual stimulus encoding we compared decoding accuracy in the three conditions containing visual stimuli (AV congruent, AV incongruent, V) for V1-3. Decoding performance did not differ significantly between conditions in V1 (*F*_(2,28)_ = 0.46, *p* = .64, *n.s.*). However, the presence and congruence of sounds had a significant effect on decoding performance in area V2 (*F*_(2,28)_ = 7.17, *p* = .003) and there was a non-significant trend for such an effect in area V3 (*F*_(2,28)_ = 2.12, *p* = .14, *n.s.*). Post-hoc t-tests revealed that stimulus decoding from activity patterns in area V2 was significantly worse in the AV incongruent condition compared to both, decoding in the AV congruent (*t*_(14)_ = 3.29, *p* = .005) and V (*t*_(14)_ = 3.46, *p* = .004) conditions. Pattern decoding from area V3 was significantly worse for the AV incongruent condition compared to the V condition (*t*_(14)_ = 2.15, *p* = .049).

To further investigate the effect of sounds on stimulus decoding from activation patterns in V1-3 we compared the reliability and similarity of stimulus-evoked patterns (cf. Materials and methods for details). There was no detectable influence of sounds on pattern similarity in V1-3 (V1: *F*(2,28) = 0.762, *p* = .476, *n.s.*, V2: *F*(2,28) = 1.069, *p* = .357, *n.s.*, V3: *F*(2,28) = 1.815, *p* = .181, *n.s.*; cf. Fig. 2d). However, pattern reliability was significantly affected by the presence of sounds in V2 and V3 (V1: *F*(2,28) = 2.013, *p* = .152, *n.s.*, V2: *F*(1.4,28, Greenhouse–Geisser corrected) = 6.647, *p* = .011, V3: *F*(2,28) = 5.133 *p* = .013; cf. Fig. 2c) Post-hoc paired t-tests revealed that pattern reliability in V2 was significantly reduced in the AV incongruent condition, compared to both the AV congruent condition (*t*_(14)_ = − 2.376, *p* = .032) and the V condition (*t*_(14)_ = − 5.406, *p* < .0001). Pattern reliability in V3 was significantly reduced in the AV incongruent condition, compared to the V condition (*t*_(14)_ = − 3.004, *p* = .010).

For completeness, we computed a complete representation of all possible stimulus pattern correlations (16 by 16); please see the Supplementary results and Fig. S1.

Our study was limited to investigating multisensory modulation of pattern discriminability in early visual cortices. It would have been interesting to compare this to similar modulations in early auditory cortex. However, auditory pattern decoding from BOLD signals typically has much lower accuracies than visual pattern decoding and appears to require high spatial resolution MRI sequences (e.g. Formisano et al., 2008; Staeren et al., 2009). Nevertheless, for completeness we also extracted patterns of BOLD signals from bilateral anterior transversal temporal gyri (Destrieux et al., 2010) and tried to classify them. Stimulus decoding was generally unsuccessful for this data and did not improve even when using a more lenient anatomical criterion (including the whole of the superior temporal gyrus and plane). We conclude that an investigation of primary auditory cortex similar to our visual cortex analysis would rely on high-resolution scans and adequate functional localizers, ideally tonotopic-mapping.

#### Searchlight results

We tested three contrasts: AV congruent–AV incongruent, AV congruent–V and V–AV incongruent (see Materials and methods for details.).

**Fig. 3:**
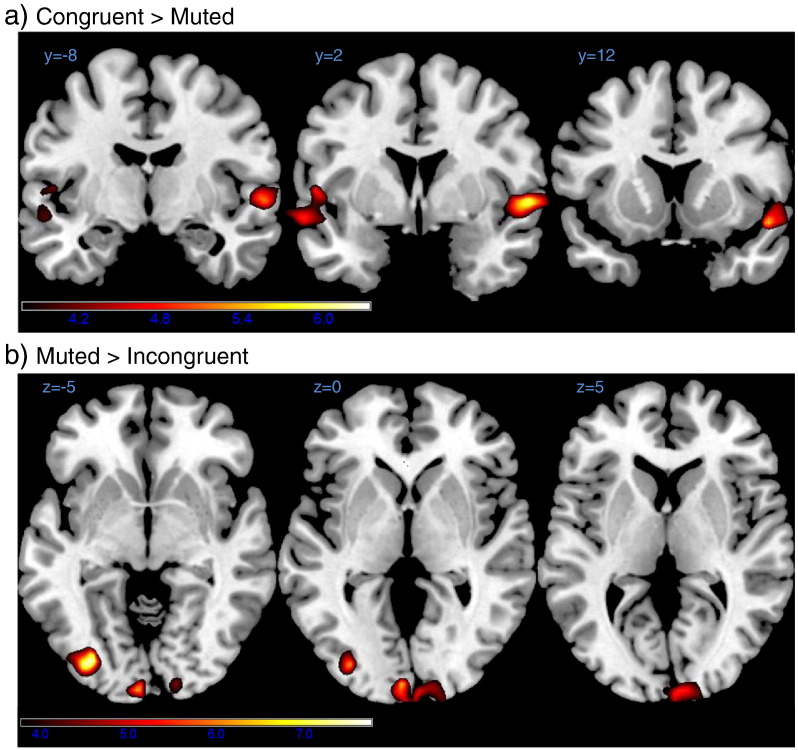
Results for whole brain searchlight analysis. Heat maps for searchlight contrasts. Searchlight maps indicating local pattern discriminability for each condition were normalised and contrasted on the second level (see Materials and methods for details). Colour coding for *t*-values is indicated by colour bars at the bottom of a) and b). Please note that the contrast between the audiovisual incongruent and congruent conditions was tested as well but yielded no significant results. Note that contrasts are directed and that contrasts of opposite direction yielded no significant results. a)Increased pattern discriminability for the audio-visual congruent condition as compared with the visual only condition in bilateral superior temporal gyrus (see Table 1 and Results section for details).b)Increased pattern discriminability for the visual only condition as compared with the audio-visual incongruent condition in left lateral occipital area and the banks of the calcarine.

The AV congruent–AV incongruent contrast yielded no significant clusters at the corrected threshold. The AV congruent–V contrast revealed two significant clusters in the bilateral superior temporal gyri (FWE corrected *p* < .05). Both clusters included early auditory cortex and part of the superior temporal gyrus (including TE 1.0, 1.2 and 3) and the right cluster extended in anterior direction to the temporal pole (cf. Table 1 and Fig. 3a)). The V–AV incongruent contrast yielded two significant clusters in visual cortex (FWE corrected *p* < .05). The first cluster spanned part of the bilateral calcarine gyrus near the occipital pole, including parts of Brodmann area 17 and 18. The second cluster was located in the left lateral inferior occipital gyrus and coincided with the location reported for areas LO1/2 (Larsson and Heeger, 2006). See Table 1 and Fig. 3b).

### Univariate fMRI analysis

To test where in the brain auditory context modulated the amplitude of the signal evoked by our stimuli (as opposed to information carried), we employed a univariate whole brain analysis. We tested the same three contrasts tested in the searchlight analysis: AV congruent–AV incongruent, AV congruent–V and V–AV incongruent (see Materials and methods for details).

**Fig. 4:**
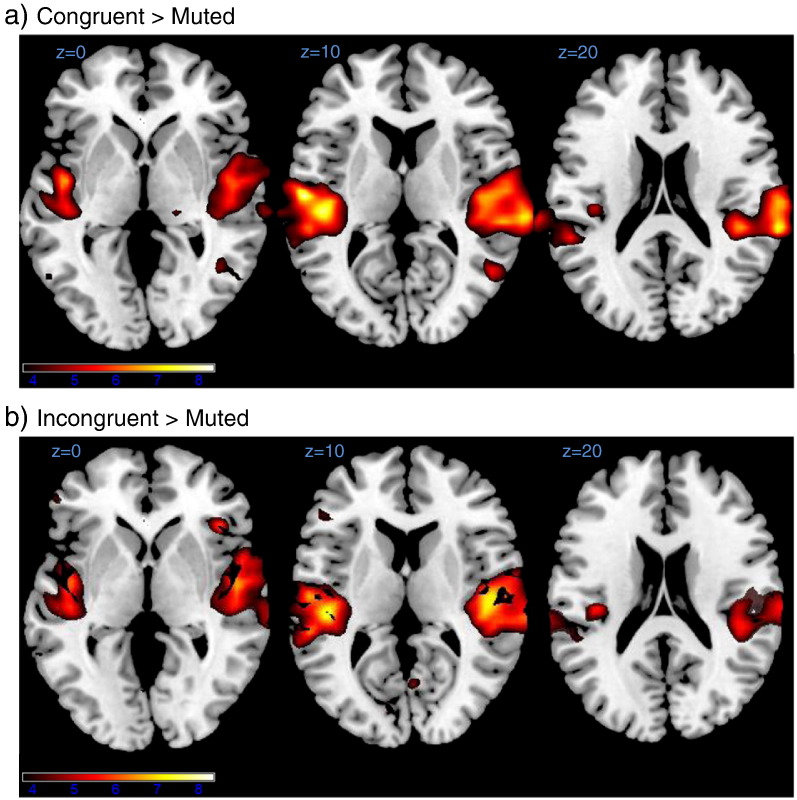
Results for whole brain univariate analysis. Heat maps indicating differences in signal amplitude between conditions. Colour coding for *t*-values is indicated by the colour bar at the bottom. See Results section and Table 2 for details. Note that contrasts are directed and that contrasts of opposite direction yielded no significant results. a)Increased signal amplitude for the audio-visual congruent condition as compared with the visual only condition in bilateral superior temporal gyri.b)Increased signal amplitude for the audio-visual incongruent condition as compared with the visual only condition in bilateral superior temporal gyri.

The AV congruent–AV incongruent contrast yielded no significant results. The AV congruent–V contrast yielded two significant clusters in the bilateral superior temporal gyri (FWE corrected *p* < .05). Both clusters included early auditory cortex (including TE 1.0, 1.1, 1.2 and 3) and the right cluster extended in anterior direction to the temporal pole (cf. Table 2 and Fig. 4a), note the similarity to the corresponding searchlight contrast). The V–AV incongruent contrast yielded two similar clusters of significantly greater activation for the AV incongruent condition (i.e. the one including auditory stimulation). These clusters again spanned almost the whole of bilateral superior temporal gyri, including early auditory cortex (cf. Table 2 and Fig 4b).

For a more direct comparison between univariate contrasts and the multivariate analysis we also tested for univariate effects in the retinotopically defined ROIs of each participant. For this contrast we averaged the voxel responses (betas) for each participant and condition across the whole of the respective ROI (cf. Fig. 2b)). Response amplitudes did not differ significantly between the three conditions involving visual stimuli in all three ROIs (V1: *F*_(2,28)_ = 0.01, *p* = .99, *n.s.*; V2: *F*_(2,28)_ = 0.25, *p* = .78, *n.s.*; V3: *F*_(2,28)_ = 1.12, *p* = .34).

## Discussion

We presented participants with naturalistic, dynamic audiovisual stimuli while they performed an incidental fixation task. Replicating previous studies (e.g. Nishimoto et al., 2011), we could decode stimulus identity from spatial patterns of BOLD signals in retinotopic cortices well above chance. More specifically, we could decode stimulus identity significantly better than chance from BOLD patterns in V1-3 (separately) for all conditions containing visual stimuli (AV congruent, AV incongruent and V), but not for the audio only (A) condition.

There were no detectable differences in mean amplitudes of BOLD signals evoked in V1-3 for the AV congruent, AV incongruent and V conditions. However, and most importantly, decoding accuracy varied significantly with the presence and congruence of sounds in V2 and somewhat in V3. Decoding accuracy for patterns in V2 was worse for the AV incongruent condition compared to both, the V and AV congruent condition. Decoding accuracy in V3 was worse for the AV incongruent compared to the V condition. Worsening of local decoding accuracies for the AV incongruent (compared to V) condition was confirmed and extended to area LO (and possibly V1) by searchlight analyses.

Significantly worse decoding for the AV incongruent condition in V2 (compared to the AV congruent and V conditions) was associated with reduced inter-trial reliability of patterns for a given stimulus in this condition (again, in comparison to the AV congruent and V conditions). In V3 reduced decoding accuracy for the AV incongruent condition relative to the V condition went along with reduced inter-trial reliability for the same comparison. In contrast to the reliability of *intra*-stimulus patterns, no significant modulation of *inter*-stimulus pattern similarity could be found.

### Modulation of pattern discriminability

Our results demonstrate modulation of stimulus evoked pattern discriminability as a consequence of multisensory interactions in human early retinotopic cortex. They are in accord with and extend recent findings in macaque primary auditory cortex (Kayser et al., 2010) and superior temporal sulcus (Dahl et al., 2010). Notably, we observed these modulations in early visual cortex using high-contrast visual stimuli that covered only central parts of the visual field (< 10° eccentricity). Our data suggest that this effect reflected modulations of inter-trial reliability of neural activation patterns for a given stimulus, i.e. the average multivariate mean for a given stimulus was not shifted, but the trial-by-trial scatter around this mean depended on multisensory context. This is also in line with the findings of Kayser et al. (2010) and Dahl et al. (2010).

Note that we could not discriminate BOLD signal patterns in visual cortex evoked by purely auditory stimuli. This contrasts with the findings that auditory motion direction can be decoded from lateral occipital cortex (Alink et al., 2012) and visual stimulus identity can be decoded from early auditory cortex (Hsieh et al., 2012; Meyer et al., 2010). A possible explanation for this difference is that such effects rely on top-down attention or even cross-modally evoked imagery (Hsieh et al., 2012; Meyer et al., 2010). It is possible that this kind of effect was prohibited or attenuated by our fixation task. Alternatively, it is possible that only certain types of auditory signal such as those associated with motion can be decoded from visual cortex.

Interestingly modulations of BOLD pattern discriminability in visual cortices were not accompanied by overall amplitude modulations in our experiment. This differs from the results of previous fMRI studies that found increased univariate signals in early sensory areas for audiovisual concurrent compared to purely visual stimulation (e.g. Martuzzi et al., 2007; Noesselt et al., 2007; Watkins et al., 2006). This difference might reflect the fact that these earlier studies used transient, periliminal or low contrast stimuli while here we used naturalistic stimuli. Also, Kayser et al. (2010) and Dahl et al. (2010) found some net amplitude *reduction* for bimodal stimulation. However, our V condition differed from their design: in our experiment it was not truly unimodal because scanner noise was present throughout the experiment. Increased BOLD amplitude is also observed in parts of early visual cortex for spatially incongruent (vs. congruent) audiovisual stimuli (Meienbrock et al., 2007). Our failure to find such an effect might be due to differences in stimuli and design. Audiovisual in/congruence was specific to spatial alignment in that earlier study while our manipulation affected temporal and semantic congruence as well. Also, we used an orthogonal fixation task, while the earlier study required participants to explicitly judge the spatial congruency of stimuli. Congruency effects may therefore be task-dependent and this should be examined in future work. Stimulus and congruency directed attention might influence multisensory modulation of univariate response levels. Finally, the effect reported in that earlier study was only observed for a subgroup of vertices within retinotopic ROIs of one hemisphere at a relaxed statistical threshold so our failure to observe such moderate effects may be due to a lack in statistical power. Whatever the reasons for the dissociation between modulation of overall amplitude and pattern discriminability in the present work, it renders our results important in the context of the debate about criteria for multisensory interactions. These usually concern different types of amplitude modulation and the question which of them qualify as ‘multisensory’ (e.g. Beauchamp, 2005). Our results demonstrate multisensory interactions in the absence of *any* detectable net amplitude modulation. Furthermore, one might argue that, in the context of naturalistic stimuli, modulation of pattern discriminability may be the most relevant effect of multisensory interactions. Recently, it has been argued that the role of primary sensory cortices in audio-visual integration might be limited to low level stimulus features and transient stimuli (Giani et al., 2012; Werner and Noppeney, 2010). The basis for this argument is the observed insensitivity of the (univariate) BOLD signal amplitude in primary auditory cortex to higher order stimulus congruence (Werner and Noppeney, 2010) and the absence of cross-modulation frequencies for audio-visual steady-state responses in MEG (Giani et al., 2012; note that the latter method does not allow the presentation of audio-visual congruent stimuli). Our results suggest the null results in these studies could reflect an insensitivity of the analysis methods used to detect modulations of the encoded stimulus information (like pattern discriminability or pattern reliability). This underscores the need for further research to clarify the exact role of primary sensory cortices in audiovisual stimulus integration.

### Potential mechanisms modulating audiovisual pattern discriminability

How do sounds affect the reliability of early visual cortex signals? Most likely this effect rests on subthreshold modulation of visual neurons, rather than on classical bimodal neurons. Bimodal neurons in early visual cortex seem to be restricted to the far periphery of visual space (which we did not stimulate here) whereas subthreshold modulation also affects more central representations (Allman and Meredith, 2007). Furthermore, multisensory modulation of spike train discriminability is found for subthreshold modulation of visual neurons (Dahl et al., 2010). One could speculate that this subthreshold modulation in turn could be mediated via phase alignment of ongoing oscillations (e.g. Lakatos et al., 2007; Naue et al., 2011; Romei et al., 2012). Some results from a recent MEG study are of particular interest (Luo et al., 2010), showing that accuracy of decoding video stimuli from phase patterns of occipital channels depends on audiovisual congruency. Furthermore, in that MEG study the trial-by-trial phase coherence (i.e. reliability) for a given video stimulus was affected by audiovisual congruency as well. It has been proposed that temporal profiles of neural activity in different primary sensory areas can work as oscillatory attractors on each other, effectively yielding an ongoing modulation of excitability (Lakatos et al., 2009; Schroeder et al., 2008). This could serve to minimise temporal uncertainty (Friston, 2009) and would be very similar to what was proposed as an early theory of ‘dynamic attention’ (Jones, 1976; Large and Jones, 1999). Note, that for our design such effects would likely be stimulus driven, rather than top-down controlled — participants were engaged in a fixation task and had no incentive to concentrate on the dynamic stimuli in the background.

If temporal fine-tuning is indeed a mechanism behind our finding, it is interesting that MVPA was sensitive enough to pick it up despite the coarse temporal resolution of fMRI and the fact that decoding rests on *spatial* patterns of activation. The studies by Kayser et al. (2010) and Dahl et al. (2010) investigated modulation of single unit firing rate variability. This could translate to BOLD pattern variability, if the variance of the net population amplitude in a voxel would be modulated in effect — or at least the variance of modulatory pre-synaptic activity contributing to the BOLD-signal (Cardoso et al., 2012; Friston, 2012).

### Null results with regard to enhanced pattern discriminability and V1

Our data did not show significant modulation of pattern discriminability in V1. For V2 and V3 they only showed reduced pattern discriminability in the AV incongruent condition, but no enhancement for the AV congruent condition. Null-results need to be interpreted cautiously for several reasons. In our case, there are additional, design-specific reasons to be cautious: Multisensory interactions are generally more likely for peripheral (e.g. Allman and Meredith, 2007) and degraded (e.g. Ernst and Banks, 2002; Fetsch et al., 2012) stimuli. However, our visual stimuli were naturalistic and had high contrast, while the sounds we used were degraded due to scanner noise. Thus our design was suboptimal for evoking maximum cross-modal interaction effects and potentially biased towards detrimental effects on visual processing rather than enhancement. That said, one might expect audio-visual effects to be stronger in V2 than V1 if they rest on direct crosstalk with auditory cortex, because these connections seem to be much sparser in V1 than in V2 (Rockland and Ojima, 2003). Furthermore, Kayser et al. (2010) found enhancement of information representation in macaque A1 for AV congruent as well as for AV incongruent stimuli. However, Dahl et al. (2010) found only significant information degradation for visual neurons in the AV incongruent condition, but no significant enhancement for the AV congruent condition. In sum, it might be possible that the signal to noise ratio (SNR) of early visual responses is close to ceiling for naturalistic stimuli, and thus early auditory responses are more likely to gain from multisensory interactions. Future studies should parametrically vary the SNR of visual stimuli (or possibly both modalities) to shed further light on this question.

### Possible sources of multisensory interactions

Our data provide information about the effects of multisensory interactions in V1-3, but not about their source(s). The multisensory effects we observed could be mediated by feedback connections from multisensory cortices, by feed-forward connections from the superior colliculus and/or by direct connections between primary sensory areas (cf. Driver and Noesselt, 2008; Klemen and Chambers, 2012) for an overview). In humans, analyses of functional connectivity could provide hints regarding these possibilities (e.g. psycho-physiological interactions (PPI) Friston et al., 1997). Unfortunately, however, the optimal design requirements for MVPA are very different from those for connectivity analyses (e.g. fast event related designs to acquire many pattern examples for MVPA vs. longer task blocks for PPI). Future studies could try to combine both analysis techniques by applying both kinds of designs in one sample. This would allow testing for correlations between the individual strength of modulation with regard to information representation and with regard to connectivity.

## Conclusions

Multisensory interactions affect human visual cortex processing from its earliest stages. For naturalistic stimuli, these interactions can be restricted to reliability modulations of fine-grained patterns and thus go undetected by common univariate analyses. This calls into question the exclusivity of criteria for multisensory interactions involving net amplitude modulation. The purpose of pattern discriminability modulations is likely to enhance encoding reliability (esp. for weak stimuli), but further research is needed.

The following are the supplementary data related to this article

Supplementary material.

Fig. S1 Full correlation matrices for stimulus evoked patterns in V1-3.
Each cell represents the correlation between two average patterns of activation. Correlations on the diagonal represent correlations of the average pattern of activity for a given stimulus in odd runs with the average pattern for this stimulus in even runs. All other averages are across all runs. Letters indicate stimulus identity (‘F'rog, ‘K'eys, ‘R'ooster, ‘P'aper), following a ‘visual/auditory’ convention (cf. Supplementary results for further explanation). Coloured rectangles indicate within-condition correlations. The colour bar to the right indicates Pearson's correlation coefficient. a) to c) represent pattern correlations for V1-3, respectively.

## Figures and Tables

**Table 1:** Significant searchlight clusters. Details of clusters where decoding accuracy was significantly different between conditions. Coordinates of peak voxels are in MNI space, cluster size is in voxels and *p*-values are whole brain FWE corrected at cluster level, *t*-values correspond to peak voxels. Anatomical labels refer to the Juelich Histological atlas. See Materials and methods for details.

Contrast	*p* value	Cluster size	*t*-value	Peak voxel	Label
AV congruent–V	< .001	861	6.33	[62 − 2 0]	r superior temporal gyrus
[50 16 − 12]	r temporal pole
[62 20 − 12]	(Not assigned)
.006	408	5.40	[− 56 − 2 8]	l superior temporal gyrus
[− 52 4 2]	l Rolandic operculum
[− 60 6 − 10]	(Not assigned)
V–AV incongruent	< .001	699	6.93	[− 32 − 82 − 4]	l inferior occipital gyrus
< .022	303	7.75	[− 4 − 94 − 2]	l calcarine bank

**Table 2:** Significant clusters for the univariate analysis. Details of clusters for which signal intensity was significantly different between conditions. Coordinates of peak voxels are in MNI space, cluster size is in voxels and *p*-values are whole brain FWE corrected at cluster level, *t*-values correspond to peak voxels. Anatomical labels refer to the Juelich Histological atlas. See Materials and methods for details.

Contrast	*p* value	Cluster size	*t*-value	Peak voxels	Labels
AV congruent–V	< .001	1392	8.32	[57 − 31 13]	r superior temporal gyrus
7.81	[69 − 22 16]	″
7.69	[54 − 7 − 8]	″
< .001	900	8.26	[− 57 − 16 10]	l superior temporal gyrus
7.76	[− 48 − 25 10]	″
7.05	[− 42 − 19 13]	l Rolandic operculum
AV incongruent–V	< .001	1461	8.04	[54 − 7 8]	r superior temporal gyrus
7.34	[57 − 31 13]	″
7.20	[45 − 19 13]	r Heschl's gyrus
< .001	1002	7.27	[− 48 − 25 10]	l superior temporal gyrus
7.17	[− 54 − 1 − 14]	″
7.02	[− 48 − 1 − 8]	″
