# Supplementary material for: Auditory modulation of visual stimulus encoding in human retinotopic cortex
Source: Neuroimage. 2013 Apr 15;70:258–67. doi: 10.1016/j.neuroimage.2012.12.061 (PMC3625122; doi:10.1016/j.neuroimage.2012.12.061)
Supplement: Supplementary file 1 — Supplementary material. [file mmc1.doc]

**Supplementary Material for ‘Auditory Modulation of Visual Stimulus Encoding in Human Retinotopic Cortex’**

**Supplementary Results**

Figures S1-3 report the results of all pair-wise correlations for stimulus-evoked patterns of activation in V1-3. Correlations between identical stimuli within the same condition (on the diagonal) were calculated as correlations between the mean patterns of BOLD signals across voxels for a given stimulus in odd and even runs. All other correlations (off-diagonal) were based on mean patterns across all runs.

Letters indicate stimulus identity with ‘F’, ‘K’, ‘R’, ‘P’ indicating the ‘Frog’, ‘Keys’, ‘Rooster’, ‘Paper’ stimuli, respectively. Letter indexing follows a ‘Visual/Auditory’ convention. So for instance ‘F/F’ indicates the video of the frog was shown with the corresponding sound track (congruent condition), while ‘F/K’ indicates the frog video was shown but the sound track substituted with the one of the keys video (incongruent condition). Letter colouring highlights specific conditions, and condition specific correlations fall within the highlighted rectangles of respective colours. Correlations from within these rectangles were averaged and used for the similarity and reliability analyses (off-diagonal and diagonal correlations, respectively).

Note the higher correlations on the diagonal vs. off the diagonal, indicating information about stimulus identity being encoded in patterns of activation. Also note the ‘mirrored’ diagonals for correlations between identical visual stimuli in the different conditions, indicating that the mean pattern for a given visual stimulus remained similar, irrespective of multisensory context. Between conditions correlations for identical visual stimuli are likely higher than the respective within-condition correlations because fewer data was available for the latter (rendering average patterns more noisy). Note that our main interest was in changes of reliability and/or similarity across conditions. We thus decided to use all available data for each measure, rather than aiming to keep the two directly comparable with each other (by e.g. disregarding half the data for between stimulus correlations). Finally, note the absence of any clear correlation patterns for correlations involving audio-only patterns (the right angle stretching from the lower right corner).

**Supplementary Figure Captions**

**Figure S1: Full Correlation Matrices for Stimulus Evoked Patterns in V1-3**

Each cell represents the correlation between two average patterns of activation. Correlations on the diagonal represent correlations of the average pattern of activity for a given stimulus in odd runs with the average pattern for this stimulus in even runs. All other averages are across all runs. Letters indicate stimulus identity (‘F’rog, ‘K’eys, ‘R’ooster, ‘P’aper), following a ‘Visual/Auditory’ convention (cf. Supplementary Results for further explanation). Coloured rectangles indicate within-condition correlations. The colour bar to the right indicates Pearson’s correlation coefficient. a) to c) represent pattern correlations for V1-3, respectively.
