# Supplementary material for: Auditory modulation of visual stimulus encoding in human retinotopic cortex
Source: Neuroimage. 2013 Apr 15;70:258–67. doi: 10.1016/j.neuroimage.2012.12.061 (PMC3625122; doi:10.1016/j.neuroimage.2012.12.061)
Supplement: Fig. S1 — Full correlation matrices for stimulus evoked patterns in V1-3. Each cell represents the correlation between two average patterns of activation. Correlations on the diagonal represent correlations of the average pattern of activity for a given stimulus in odd runs with the average pattern for this stimulus in even runs. All other averages are across all runs. Letters indicate stimulus identity (‘F'rog, ‘K'eys, ‘R'ooster, ‘P'aper), following a ‘visual/auditory’ convention (cf. Supplementary results for further explanation). Coloured rectangles indicate within-condition correlations. The colour bar to the right indicates Pearson's correlation coefficient. a) to c) represent pattern correlations for V1-3, respectively. [file mmc2.pptx]

## Slide 1
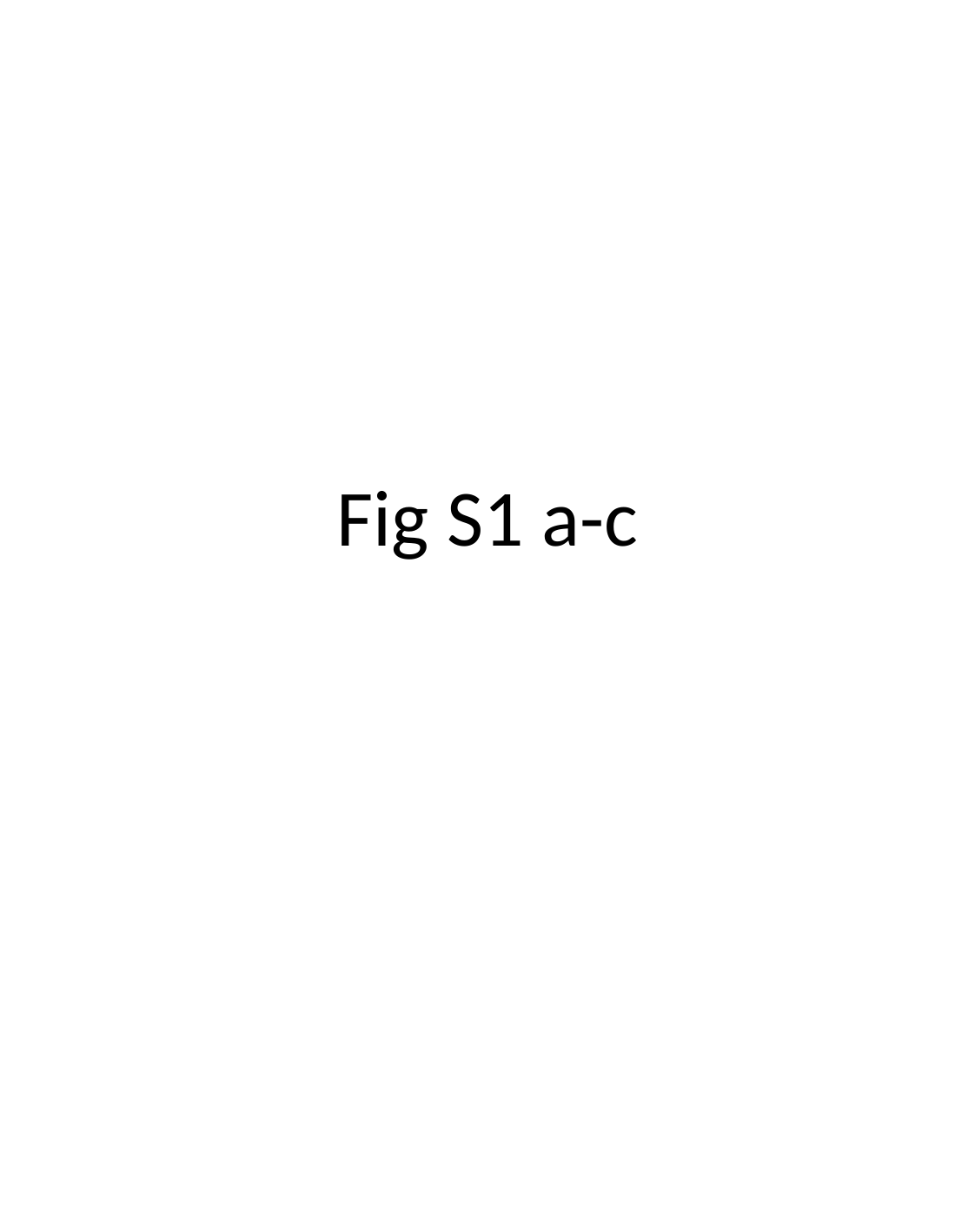

# Fig S1 a-c

## Slide 2
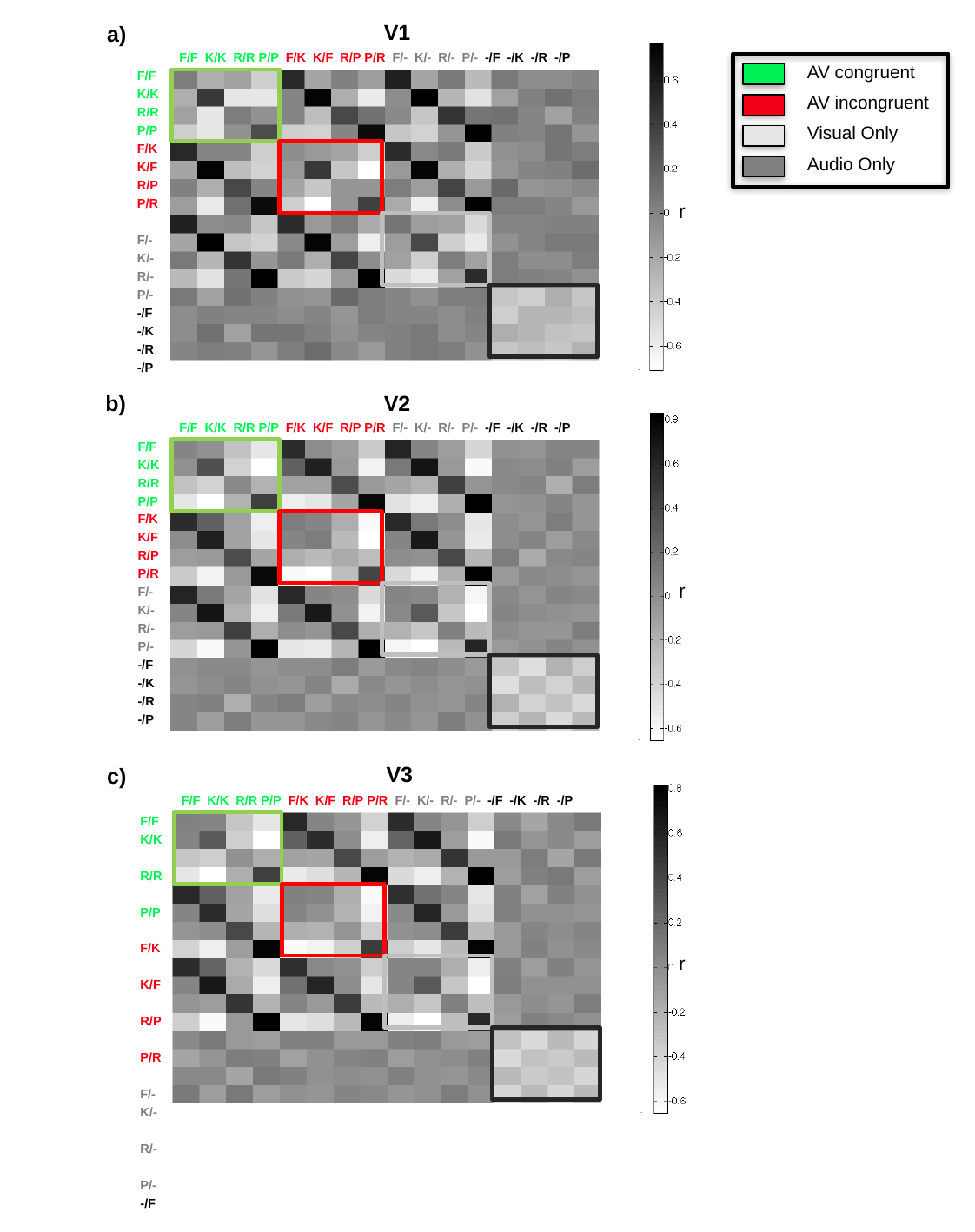

V1
F/F K/K R/R P/P F/K K/F R/P P/R F/- K/- R/- P/- -/F -/K -/R -/P
F/F
K/K
R/R
P/P
F/K
K/F
R/P
P/R
F/-
K/-
R/-
P/-
-/F
-/K
-/R
-/P
a)
AV congruent
AV incongruent
Visual Only
Audio Only
r
b)
V2
F/F K/K R/R P/P F/K K/F R/P P/R F/- K/- R/- P/- -/F -/K -/R -/P
F/F
K/K
R/R
P/P
F/K
K/F
R/P
P/R
F/-
K/-
R/-
P/-
-/F
-/K
-/R
-/P
r
V3
F/F K/K R/R P/P F/K K/F R/P P/R F/- K/- R/- P/- -/F -/K -/R -/P
F/F
K/K
R/R
P/P
F/K
K/F
R/P
P/R
F/-
K/-
R/-
P/-
-/F
-/K
-/R
-/P
c)
r
